# Patients’ perceptions of the triage system in a primary healthcare facility, Cape Town, South Africa

**DOI:** 10.4102/phcfm.v8i1.1148

**Published:** 2016-06-17

**Authors:** Adeloye Amoo Adeniji, Bob Mash

**Affiliations:** 1 Division of Family Medicine and Primary Care, Stellenbosch University, South Africa

## Abstract

**Background:**

In public healthcare facilities, where the patient numbers and the available resources are often disproportionate, triage is used to prioritise when patients are seen. Patients may not understand the triage process and have strong views on how to improve their experience.

**Aim:**

This study explored the views of patients who had undergone triage in the emergency centre of a primary care facility.

**Setting:**

Gugulethu Community Health Centre, Cape Town.

**Methods:**

A purposive sample consisted of five women (one coded green, three orange, one yellow) and four men (one coded green and three yellow). A semi-structured qualitative interview was conducted in either Xhosa or English and the transcripts analysed using the framework method.

**Results:**

All of the respondents complained of a lack of information and poor understanding of the triage process. Those coded green experienced the process as biased and unfair and reported that the triage nurse was rude and unprofessional. By contrast, those coded yellow or orange found the triage nurse to be helpful and professional. Most patients turned to support staff (e.g. security staff or cleaners) for assistance in dealing with the triage system. Most patients waited longer than the guidelines recommend and the green-coded patients complained about this issue.

**Conclusion:**

Patients did not have a good experience of the triage system. Managers of the triage system need to design better strategies to improve patient acceptance and share information. The important role of support staff needs to be recognised and strengthened.

## Introduction

South Africa is facing a quadruple burden of disease:^1^ HIV with tuberculosis (TB), increased maternal and child mortality, injuries/violence, and the growing burden of non-communicable diseases.^1,2^ The Medical Research Council of South Africa estimated 10.5% of the national mortality is due to homicide, violence, and road traffic accidents.^1^ Seventy thousand South Africans are killed by trauma and a further 3.5 million seek trauma care on a yearly basis.^3^ At the primary care level, emergencies associated with all these conditions present together at the emergency unit and place a huge demand on the services.

The District Health System has been adopted as the vehicle to deliver comprehensive primary healthcare services in South Africa.^4^ These services include acute emergency care in primary care facilities, which in the Cape Town metropolitan area is offered by the 24-hour community health centres. Many patients present at the emergency centres (ECs), from those with immediate life-threatening emergencies to those seeking basic primary care services.^3^ An effective triage system is, therefore, imperative to sort patients by need into those that must be seen immediately from those that can safely wait.

The development of emergency medicine as a speciality over the past 30 years in countries in North America, Europe, the Middle East, and Australasia has been closely associated with successful implementation of triage.^5^ The disciplines of family medicine and emergency medicine in South Africa are also championing the introduction of a triage system.

The Western Cape Health Department allocates staff and resources according to the level of need in hospital ECs.^6^ The 24-hour community health centres are structured to offer basic emergency and trauma care in the Western Cape. Primary care facilities in South Africa are generally poorly resourced, understaffed, overcrowded, and open for limited hours.^6^ Effective triage is therefore needed to ensure an appropriate utilisation of these scarce resources and personnel based on the principle of prioritisation.

The modern day evolution of triage is conceptualised around scarcity of healthcare resources, including personnel.^7^ Triage is deemed pointless where there is no scarcity of medical resources or where there are no resources available.^7,8^ The five–level EC triage system is adopted for use in the United States, Canada, Spain, the United Kingdom, and Australia. Several different triage systems are described such as the emergency severity index,^9^ the Manchester Triage scale,^10^ and the Canadian Triage and Acuity scale.^11^ None of these methods have been proven to have an excellent inter-rater reliability.^8^ However, all of them have been shown to be very important in overall patient flow and prioritisation.^9,10,11,12^ Therefore, triage is an internationally recognised approach to patient management based on principles of distributive justice.

The South African Triage Group (SATG) is a designated triage body with a focus on improving equity and quality in emergency healthcare.^13^ The operational manual of the SATG is the South African Triage Scale (SATS). SATS was rolled out in 2008 in three versions: adult, child, and infant.^13^ The aims and goals of the SATS are ‘to expedite the delivery of time-critical treatment for patients with life-threatening conditions, to ensure that all people requiring emergency care are appropriately categorised according to their clinical condition, to improve patient overall length of stay, to facilitate streaming of less urgent patients and to be user friendly for all level of health care professionals’.^5^ SATS is a simple and user friendly tool appropriate to the South African healthcare system.^8^ Patients are categorised into red, orange, yellow, green, or blue (dead) based on their triage scores. The triage score is based on their vital signs and a list of specific emergency conditions. The approximate expected waiting time is mostly determined by the colour category of the patient. The red patients are to be seen immediately, orange to be seen in less than 10 minutes, yellow to be seen in less than 60 minutes, and green to be seen in less than 240 minutes.^5^ Patients who have waited longer than their expected waiting time are supposed to be ‘re-triaged’; however, re-triage is a concept that has been very difficult to implement in the South African setting. The major focus of triage is the patient;^14^ therefore, their perceptions of the process is a very important index of measuring the success of this system. Patient satisfaction has become an increasingly important concept in healthcare.^15^

Research elsewhere has evaluated the relationships between patients and their triage experiences,^14,15,16,17^ and a key measure of a positive experience is the patient’s willingness to return. The three major factors that are important in determining patients’ satisfaction are interpersonal skills/staff attitudes, provision of information/explanation of reason for waiting, and perceived waiting time.^17^ It was observed that the provision of information goes a long way to influencing patient perceptions of the triage system.

This study therefore aimed to explore the ethical, social, and operational issues surrounding triage by exploring the patients’ viewpoints and experiences of triage in the EC at Guguletu Community Health Clinic (CHC), Cape Town.

## Methods

### Study design

This was a qualitative study using semi-structured interviews with key informants.

### Setting

This research was conducted at Gugulethu CHC, a 24-hour primary care facility located in a predominantly Xhosa-speaking, economically disadvantaged township, about 18 km from the centre of Cape Town. The majority of the youth are unemployed or unemployable due to a lack of necessary skills and education to compete in the open labour market. Most of the residents therefore rely on the CHC as the only source of healthcare.

On arrival at the EC, patients open their folders at reception and then leave their folders at the security desk while waiting to be called in for triage. The triage waiting room is small and separated from the EC by an iron barrier gate manned by a security guard. The first room, just after the gate, is the triage room. Triage is performed by a trained enrolled nursing assistant. Patients are called in to the triage room, assessed and colour-coded, and then go back to the waiting room. Patients therefore have to wait three times during this process – firstly to open a folder, secondly to be triaged, and finally to be consulted.

### Study population and sampling

Our initial approach was to randomly select patients from the EC attendance register, spread across the colour codes (excluding patients coded red), who had attended the EC in the previous 2 months. We tried to contact these patients via their registration profiles (phone numbers). Unfortunately, only two patients were recruited through this means and the majority had provided invalid phone numbers or were not interested in the study. Therefore a further seven participants were recruited during their actual visit to the EC and again were selected from different colour categories, from different genders, and from both office hours and after-hours. It was assumed that all adult patients would have a useful perspective on their experience and therefore could be selected. Every alternate folder in each colour category was selected and the patients were contacted after they had been attended to by the clinician and invited to participate in the study.

The study was fully explained to the participants in isiXhosa and written consent was obtained. The initial analysis ran concurrently with each stage of recruitment, and no new themes emerged by the time the ninth participant was interviewed.

### Data collection

The majority of the participants were interviewed in isiXhosa, which is the predominant language in the community; one participant felt comfortable being interviewed in English. Semi-structured interviews were conducted based on an interview guide (Appendix 1). The interview was opened with the question: ‘Can you tell us everything that happened to you from the time you arrived at the clinic until you saw the doctor?’ Patients’ responses usually gave direction on the areas to explore in more depth without losing the focus of the interview. The other questions were directed towards exploring the patients’ experiences of the waiting room, triage room, staff attitudes, and information offered. After the initial interview, a 5-minute DVD was played that explained the basic concepts of triage. Participants were then asked again how they felt about the process they had gone through.

The interview was conducted in a quiet and private consulting room by a isiXhosa-speaking interviewer who was trained in qualitative interviewing. The interviewer received 2 hours of training to understand the triage system and orientate her to the functioning of the EC. Author AA ensured that he was available for every interview to support the interviewer should any questions arise. The responses to the interview were audio-recorded. A summary of the responses was validated with the interviewees by phone call, and this contact also enabled any further clarification of their viewpoint.

### Analysis

The audio recordings of the interviews were translated into English and transcribed verbatim by the Xhosa interviewer. The resulting manuscript was proofread and corrected for grammatical errors by the researcher and the interviewer. The framework method was used for analysis; it consisted of five steps:^18^

*Familiarisation:* The researcher familiarised himself with the transcripts and noted key ideas as well as any issues with the quality of the data.

*Formation of thematic framework:* The researcher defined codes and organised them into categories in order to develop a thematic index or framework. Codes were derived inductively from the transcripts and the categories were aligned with the objectives of the study.

*Indexing:* The codes were then applied to all the transcripts using Atlas.ti software.

*Charting:* Charting brought all the data related to a particular code or category together in the same place. Atlas.ti was used to facilitate the creation of charts.

*Interpretation:* The charted data were studied, analysed, and interpreted in order to identify the nature and range of different opinions and any associations or relationships within the data.

## Ethical considerations

The research was approved by the Health Research Ethics Committee of Stellenbosch University (ref. S13/08/151) and permission to use the Gugulethu CHC was obtained from the Provincial Department of Health in Western Cape.

## Results

Nine participants were involved in the study. In terms of the colour code, two were coded green, four yellow, and three orange. There were five women, of whom one was the mother of a child triaged yellow, and four men. Participants’ ages ranged from 19 to 68 years. The participants include patients seen during the working hours as well as patients who had their consultation during the extended hours.

### Patients’ expectation of primary care

Patient’s expectations of the health centre were partly determined by the way in which they accessed care. One group of patients had chosen to attend the health centre, while the other group was brought to the health centre by emergency medical services. The patients who had decided for themselves to seek care at the health centre expected timely, efficient, quality care and to be referred onwards if necessary:

‘As I told you, I was so sick that I had to call an ambulance, so I was thinking I would be attended to immediately once I got there, and possibly be transferred to GFJ Hospital for better treatment.’ (Female patient coded orange)

Patients brought to the health centre by emergency medical services would often have preferred to be taken elsewhere and had low expectations of the service. Nevertheless, some of them expressed surprise about the type of healthcare they could access at the health centre:

‘I was expecting to receive a very ugly treatment from the staff because there was this widely held view in the community that KTC is a bad place, but I was surprised because the view was wrong and unfounded.’ (Male patient coded yellow)

Some patients expected a triage waiting area in which different categories of patient were separated and, therefore, expressed disappointment when they found out that medical, trauma, and paediatric patients were all put together:

‘So I think they must renovate the unit so that there can be more rooms to separate different categories of patient. Maybe in that way they will be able to sort out the people in the right way.’ (Female patient coded orange)

Most of the patients expected a larger health centre, with increased resources and personnel to cope with the fast-growing community of Gugulethu:

‘I think if the government can employ more doctors and nurses and cleaners and security and possibly build a bigger hospital for us in Gugulethu, then everything will work well for all of us in this community.’ (Male patient coded yellow)

We can, therefore, see that overall patients’ expectations revolved around waiting time, quality, and acceptability of healthcare provided and the infrastructure.

### Information about and patients’ understanding of the triage system

Patients’ understanding of the triage system was informed either by their prior experience of triage, the information offered by the healthcare workers, or information gathered from other sources, such as posters, in the course of triage.

The majority of the patients did not know anything about the triage system and had widely divergent views on who decides and how they decide on who should be seen by the clinician and at what time. Even the few more regular users, who knew there was a process of sorting patients, were unclear about how it works:

‘No, I didn’t know anything about triage before I was invited for this study.’ (Female patient coded green)

‘Oh, I was treated as if the information about me and my health was actually not my right. Everybody in the room was waiting without knowledge of how the system works.’ (Female patient coded orange)

‘No, I did not know. I am not usually sick, so I don’t know a lot about hospital systems, like this triage.’ (Male patient coded yellow)

Most of the patients expressed disappointment about the lack of information obtained from the healthcare workers. Even those patients that commented positively about the healthcare services rendered were not happy about the level of information offered to them about the triage system. The information deficit included a lack of information about how sick they were thought to be after being triaged and how they had been coded, being uninformed about the reasons for or results of blood and urine test done during triage, and not knowing the approximate waiting time expected. Some patients even misinterpreted the basic triage investigations and believed that an HIV test was being done without their consent. The lack of information was framed as a denial of their rights as patients and clearly undermined their experience and trust in the triage system:

‘It was my first time at the clinic so I didn’t know what was happening; like, was she taking HIV test or what? I didn’t know because she was not explaining anything to me. I think it would have been better if she told me every step that she was going to do with me. This is my first time in this clinic and I don’t know how the system works, but I think every system in South Africa respects the right of every citizen to adequate information.’ (Male patient coded yellow)

‘I think this is information that the clinic should take serious, they should make sure that everybody waiting to be attended to understand this colour system, so that nobody will complain. I also think every patient should know their colour so that they can complain if someone from their colour who arrived after them is being attended before them.’ (Female patient coded green)

‘No information was given to me.’ (Female patient coded orange)

‘Lack of information is a very big deficit in this clinic. Everybody is treated as if you know everything happening in the system.’ (Male patient triaged yellow)

The provision of information through posters on the walls of the facility was not regarded as adequate and most patients were unaware of any relevant information on triage given in this way. Most patients, therefore, suggested the appointment of a designated information officer or use of television in the triage waiting room to properly inform them about the triage system:

‘I think if there can be someone who explains everything to us like: What is going to happen? Why are you sitting here? Like an educator, or if there can be TVs that are showing these adverts to the people. But still there must be someone who must explain everything that they are saying in the TV; alternatively, they can make the video to be played in Xhosa since everybody in this community understands Xhosa. Everyone can then have an understanding of what is going on, even people who are not educated. In that way, people will understand why they have to wait for so long sometimes before they see the doctor or sometimes why you may not even see the doctor. But if someone explained that to you, then you wouldn’t mind because you would know maybe you were not so sick, but you can still come back the next day to see the doctor. People panic because they don’t know what is going on, like I said all the people who were seen before me knew the nurses. But if I knew how the system worked, I would not say that. The nurses must just explain all the process to us; it’s their job to do that.’ (Male patient coded yellow)

It was clear that there was a gross information deficit and a lack of understanding of the triage system in the facility.

### Patients’ experience of the triage process

The waiting room accommodated different categories of patients who were there for different purposes: patients yet to be triaged, patients triaged and awaiting consultation, patients who were looking for information on how the system works, patients who were waiting for transfer to other facilities, patients who were waiting for review by clinicians, and patients who were under temporary observation. The majority of patients described the waiting room as inefficient, unhealthy, and noisy. These patients complained of being exposed to stressful and disturbing scenes, and some of them felt that their exposure to the experience could worsen their own clinical condition:

‘To me; I think I have a problem with mixing everyone in the same room (children, people like me with sugar problems and people with blood all over them were kept in the same room), this is scary to me, I am not used to seeing human blood, but I had to endure this because I need to be taken care of.’ (Female patient coded orange)

As most patients were unaware of the rationale behind the triage system and lacked information about what was happening, they interpreted the process as being unfair or biased and believed that nurses were simply rearranging patients according to whether they knew them or not. In this context, patients felt strongly that it was fairer for patients to be seen in the order of their arrival:

‘I don’t think that it is fair, because everyone who comes to the clinic comes in very early so that you can go home in time. So if you sit here the whole day just because there are people who know the nurses, who can help them before you, it is not fair. We must be helped the way we came in; if I was the first to get here I must be the first to see the doctor. Unlike today, some other days are so annoying because the nurses rearrange patients according to who they know.’ (Mother of sick child coded yellow)

‘I felt so bad because I waited for a very long time and there were people who were here after me and they helped them first. So I felt guilt but I told myself that I will wait.’ (Female patient coded green)

‘I do not think it was fair; we should be attended to on a first-come, first-served basis. A lot of patients who came after me were seen before me.’ (Female patient coded orange)

Many of the patients were not happy with the amount of time it took waiting to be consulted by a clinician; however, they all seemed to see this as part of the experience they must pass through to access healthcare in the primary healthcare facility. This has therefore become a normative experience for most of the patients. Respondents who reported short waiting periods actually stayed far longer than the internationally acceptable time for their colour code categories:

‘It was about an hour or more, but it was not too long. But I stayed because I came here around 7, then they attended to me at 9 o’clock. So I think it was about 2 hours. Not a long waiting time, judging from my previous experiences.’ (Female patient coded orange)

### Patients’ experience of healthcare workers

Patients appeared to experience the healthcare workers differently depending on whether they were coded green or yellow/orange. The green patients perceived the healthcare workers as being harsh, rude, and unprofessional. They believed that the triage workers were necessary evils that must be overcome in their journey to see the doctor. They therefore saw the triage officers as people that must be tolerated in order to access the healthcare system:

‘The only thing I can say and my experience there is that they don’t care; but you have to be careful not to express your own emotions so that they can help you.’ (Female patient coded green during after-hours)

The two patients coded green reported being ridiculed by the triage officers, because they were presenting with illnesses that were not so serious in an EC during the weekend. Triage officers were seen as powerful obstacles to care who decide on who gets to see the clinician:

‘I told them that I have chest pain and a headache. They told me that they don’t help people with those problems on weekends; they only help people with critical conditions. I was warned not to do that again; I must only come during the week for conditions like that. So I asked the nurse whether I should go back home; she said I must go and sit outside, that the doctor would call me later. I sat there for a very long time till the doctor called me.’ (Female patient coded green during after-hours)

The patients coded yellow or orange, however, expressed an opposite perception of the healthcare workers. The majority of them experienced a caring, respectful, dutiful, and highly professional triage officer. They reported a friendly and positive encounter with the healthcare workers. All of them felt that the triage officers were always willing to help:

‘The nurse called me and examined me, took my urine, and tested my urine and my blood pressure; there were no problems, not the way that I have expected. The service was good.’ (Male patient coded yellow)

‘The nurses were fine like I said; if they can just tell the people what is going on, then everything will be fine. They are nice and OK but they didn’t explain all the processes and system to the people.’ (Male patient coded yellow)

A few of the yellow patients still complained that they were not fully informed about the triage process:

‘Yes, they were willing to help me and they were respectful, although they didn’t tell me anything after that or they didn’t explain anything to me, but they did their job.’ (Female patient coded yellow during after-hours)

### Patients’ experience of support staff

Two categories of support staff, the security officers and the receptionists, were also identified to have a very important influence on the patients’ overall triage experience.

The majority of patients reported being spontaneously helped by the security officers or receiving help when they requested it. This help included being assisted with a wheelchair, giving directions to help them in the process of obtaining folders, and giving information about the triage system (which was not necessarily correct). Patients felt free to approach and express themselves to the security staff, more so than with the healthcare workers:

‘OK, my name is SM. When I got to the gate, the security guard brought me a wheelchair because I could not walk. Then he pushed me from the gate to the trauma department. The other security guard said I needed to open a folder to be seen.’ (Female patient coded orange)

Some patients were content with the process of opening or obtaining a folder in the reception area. They were happy that they were given information on how to open the folders and they felt that the triage system in the EC should follow the same trend:

‘Like when I was in the reception, they did well to let us know that they would open folders for the children first, followed by people with cards, then followed by newcomers.’ (Male patient coded green)

A few patients, however, reported that the receptionists were rude and insensitive:

‘The only thing I can say is that the people in the reception area were very rude with people. They don’t know how to treat a person, which is the only thing I saw today. But the nurses were good.’ (Mother of child coded yellow)

These observations place the support staff in a very important position in the overall triage process in Gugulethu CHC; however, whether they have the necessary training for what they are doing or whether they are doing it correctly is the question left unanswered.

### Patients’ overall satisfaction

Patients appeared to fall into three groups in terms of their overall satisfaction with the triage system – totally satisfied, partly satisfied, or unsatisfied.

Only a minority of the respondents were completely satisfied with their experience of the triage system at this visit. Satisfaction was relative to expectations, and most of these patients were satisfied because their previous negative experiences did not recur during this visit:

‘There is nothing negative I can say about the way I was helped today, because usually we will sit here for a long time or you will get someone who is very rude to you; but today everything went well. Even the second nurse was very friendly. I liked the way they treated me today.’ (Mother of child coded yellow)

Most of the patients reported some dissatisfaction with their experience, such as a lack of information from the triage nurses, inappropriate staff attitudes, untidy and unhealthy triage waiting area (some reported being made sicker with the blood all over some patients and some patients with face masks), and unhappiness with the colour coding system:

‘Everything is fine, but my only concern is when they don’t tell people how the system works.’ (Male patient coded yellow)

‘The trauma area makes you worse if you are sick, because it was not neat. There is blood everywhere; it is just not a good place for a sick person at all.’ (Female patient coded orange)

‘It was noisy because some of the people waiting with us were drunk.’ (Female patient coded orange)

A few patients were unsatisfied to the point at which they would prefer not to receive care again at the health facility. In addition to the complaints outlined above, they felt that the system had failed to offer an acceptable standard of care to those that appeared critically injured. This led them to the conclusion that it was dangerous to receive care at this facility:

‘When I got here in the morning, I saw people who were here yesterday who had not been helped; some of them had been stabbed and [were] in pain, but had not received any help. This type of study should alert the government about this situation, because people will definitely die when they cannot receive timely assistance.’ (Male patient coded yellow)

## Discussion

**FIGURE 1:**
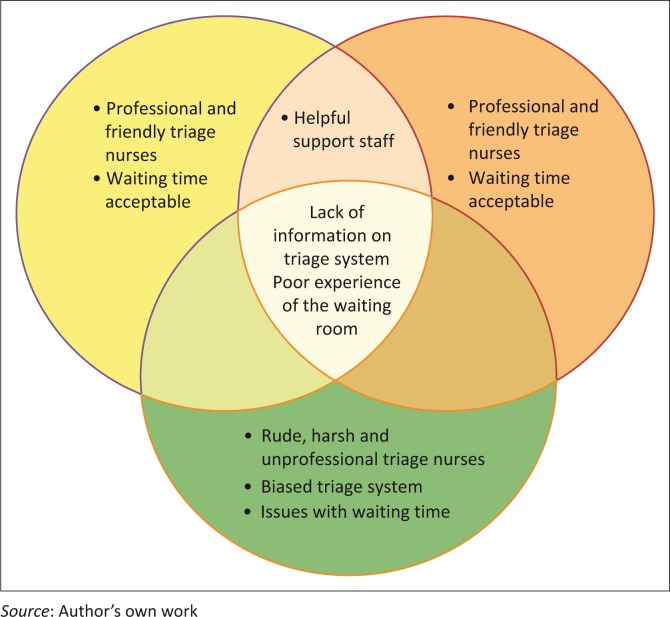
Patients’ perceptions of the triage system.

This is the first study to explore the perceptions of patients on the SATS in a local primary care setting. The key findings are summarised in Figure 1. Patients of all colour categories were united in complaining about a lack of information. They were confused about how the triage system worked and did not understand the logic or structure behind the triage process. Even those patients that commented positively about the healthcare services rendered were not happy about the level of information offered to them about the triage system.

Most of the patients described the waiting room experience as being stressful and expressed concerns about infection control. They felt that different types of patients, such as children, those with trauma, or medical cases, should be separated.

Not knowing the rationale behind triage prompted some patients to see the system as unfair and biased. Most of the patients saw long waiting times as a normative experience. Respondents who reported short waiting periods actually stayed far longer than the internationally acceptable time for their colour code categories.

The patients’ experience of the healthcare workers appeared related to their triage colour categories. The green patients had negative experiences; it appears that nurses were judgemental and blamed these patients for coming with unnecessary problems. The yellow and orange coded patients, however, reported caring, respectful, dutiful, and highly professional triage nurses.

The majority of patients reported being spontaneously helped by the security guards or receiving help when they requested it. Support staff, such as cleaners, security staff, or receptionists, may be an important and often unrecognised source of information and influence on the patients’ overall experience.

The findings of this study are consistent with other research that identified three key factors in patient satisfaction with emergency services: interpersonal skills and/or staff attitudes, provision of information and/or explanation on reason for waiting, and perceived waiting time.^17^ Actively communicating with patients about the reason they have to wait and the triage system can significantly increase patient satisfaction.^15^ Children may not be mature enough to make sense of the triage system for themselves, and in this situation communicating with the parent or caregiver is also extremely important.^19^

The relationship of colour coding to patient satisfaction is not reported elsewhere. Modifying help-seeking behaviour is one of the four core aspects of the consultation in Stott’s model^20^ and attention should be given to skills in doing this without being unprofessional, especially for those coded green. The problem may be compounded in the public setting by the lack of primary care services after-hours. In the private sector, it is much easier for patients to consult a general practitioner after working hours. In some settings, additional primary care services have been implemented in the EC to deal with non-urgent patients.^2^

National core standards for healthcare facilities place emphasis on the need to have effective infection control systems in place (*National Core Standards for Health Establishments in South Africa*, 2011). The patients’ concerns about exposure to blood and other infectious diseases such as tuberculosis appear to be real concerns. The provincial policy, *Healthcare 2030*, also places emphasis on creating a more person-centred healthcare system.^21^ This study reveals that the triage system is one area that requires attention in terms of the patients’ experiences.

### Limitations of the study

Although it appeared that saturation of themes had been reached with the nine interviews, additional interviews might have enabled more balance between colour codes, gender, time seen, and children vs. adults. The socio-cultural orientation of the Xhosa-speaking participants could affect their expectations, satisfaction, and perceptions. These issues should be considered when transferring these findings to ECs in other parts of Cape Town. The interviewer was trained and fluent in Xhosa, but was not a health professional, and therefore may not have fully understood all aspects of the health system that were mentioned by patients.

### Implications and recommendations

The following recommendations can be made from the findings of this study:

- Develop more effective systems to inform patients about the triage system and waiting times, such as use of video or electronic information boards.
- Train triage staff in more effective communication skills for modifying help-seeking behaviour.
- Recognise the important role that support staff play in the overall patient experience.
- Train support staff (such as security staff, receptionists, and cleaners) to ensure that they provide accurate information when asked for help.
- Review the infrastructure that requires all patients to wait in the same room with risks of poor infection control and psychological trauma.
- Consider alternative primary care services after working hours for patients who are coded green.

## Conclusion

The triage system at Gugulethu CHC is not adequate and patients have little information about or understanding of what is happening to them in this process. Patients coded green were treated in a judgemental and uncaring way, which led to poor satisfaction with the service. All patients experienced long waiting times relative to international norms, but those coded yellow or orange had their expectations met. Patients of all types were required to wait in the same room, leading to concerns about infection control and complaints that this was a stressful experience. Support staff, such as security guards, were a hidden resource in terms of guiding and helping patients through the system. The main recommendation is to introduce a system to inform patients more actively about the triage process and waiting times.

## Appendix 1

### Interview guide

Patients’ perceptions of triage system in a primary healthcare facility, Cape Town, South Africa.

### General considerations

1. Can you tell us everything that happened to you from the time you arrived at the clinic until you saw the doctor?
  - Can you tell us more about …?
  - What were the positive and negative things concerning …?
2. How do you feel about the amount of time you had to spend waiting to see the doctor?
3. How fair do you feel the system is in terms of who gets seen first and who gets seen later?

### Waiting room

4. What have you to say about your experience in the room where you had to wait?
  - What was the environment in the waiting room like for you as a sick patient seeking healthcare?

### Triage room

5. What have you to say about your experience in the room where the nurse asked you about why you wanted to see the doctor and took your blood pressure?
  - How long did you have to wait before the nurse attended to you?
  - After being seen by the nurse, how long did you then have to wait to see the doctor?

### Attitudes

6. What have you to say about the attitude of the staff who attended to you before you saw the doctor?

### Watch triage DVD

#### Information

7. Having watched the above DVD, how do you now feel about the process that you went through in the clinic?
  - What information was given to you to help you understand what was happening (i.e. why you had to wait and for how long you are most likely to wait)?
  - You may have noticed there were information posters in the waiting room: how much did these posters help you to understand what was happening (i.e. why you had to wait and for how long you are most likely to wait)?
  - Would you have preferred another means of getting the above information? What would have worked better for you?
  - What have you to say about the colour coding system?
  - What do you think would make this process fairer to you?

### Conclusion

8. Is there anything else you would like to say about your experience of waiting to see the doctor at the clinic?

Thank you!
